# Jiao-Tai-Wan Improves Cognitive Dysfunctions through Cholinergic Pathway in Scopolamine-Treated Mice

**DOI:** 10.1155/2018/3538763

**Published:** 2018-06-27

**Authors:** Xin-Chen Wang, Yu-Min Xu, Hong-Ying Li, Chun-Ying Wu, Ting-Ting Xu, Na-Chuan Luo, Shi-Jie Zhang, Qi Wang, Shi-Jian Quan

**Affiliations:** ^1^School of Pharmaceutical Sciences, Guangzhou University of Chinese Medicine, Guangzhou 510006, China; ^2^Institute of Clinical Pharmacology, Guangzhou University of Chinese Medicine, Guangzhou 510405, China

## Abstract

Cognitive dysfunction is characterized as the gradual loss of learning ability and cognitive function, as well as memory impairment. Jiao-tai-wan (JTW), a Chinese medicine prescription including Coptis chinensis and cinnamon, is mainly used for the treatment of insomnia, while the effect of JTW in improving cognitive function has not been reported. In this study, we employed a scopolamine- (SCOP-) treated learning and memory deficit model to explore whether JTW could alleviate cognitive dysfunction. In behavioral experiments, Morris water maze, Y-maze, fearing condition test, and novel object discrimination test were conducted. Results showed that oral administration of JTW (2.1 g/kg, 4.2 g/kg, and 8.4 g/kg) can effectively promote the ability of spatial recognition, learning and memory, and the memory ability of fresh things of SCOP-treated mice. In addition, the activity of acetylcholinesterase (AChE) was effectively decreased; the activity of choline acetyltransferase (ChAT) and concentration of acetylcholine (Ach) were improved after JTW treatment in both hippocampus and cortex of SCOP-treated mice. JTW effectively ameliorated oxidative stress because of decreased the levels of malondialdehyde (MDA) and reactive oxygen species (ROS) and increased the activities of superoxide dismutase (SOD) and catalase (CAT) in hippocampus and cortex. Furthermore, JTW promotes the expressions of neurotrophic factors including postsynaptic density protein 95 (PSD95) and synaptophysin (SYN) and brain-derived neurotrophic factor (BDNF) in both hippocampus and cortex. Nissl's staining shows that the neuroprotective effect of JTW was very effective. To sum up, JTW might be a promising candidate for the treatment of cognitive dysfunction.

## 1. Introduction

Cognitive dysfunction (CD), a central neurodegenerative disease, is closely associated with cholinergic nerve impairment. CD is defined as deficiency in memory, speech, execution, spatial discrimination ability, etc., which may possibly progress to the Alzheimer's disease (AD) [1–3]. Cholinergic neuron plays an important role in learning and memory, whose survival depends on neurite outgrowth and nerve growth factor from the target-derived phenotypic differentiation [4–7]. Another study found that positron emission tomography (PET) data show that cognitive dysfunction as the clinical stage before the onset of dementia symptoms, with the performance of cholinergic dysfunction [8]. Those opinions support the hypothesis that CD is probably an early-stage of AD, with the cholinergic nervous system affected simultaneously. Therefore, the treatment of cholinergic nervous system can alleviate the symptoms of CD and improve life quality of patients. Fujiwara et al. transplanted human choline acetyltransferase- (ChAT-) positive cholinergic neurons into the cortex of mice, which consequently alleviated the symptoms of CD by improving the cholinergic nervous system [9]. Acetylcholinesterase (AChE) inhibitors are used to treat encephalopathy by alleviating the symptoms of CD due to carbon monoxide poisoning [10]. Previous studies have confirmed that human neural stem cells overexpressing acetylcholine transferase (ChAT) play a role in the recovery of cognitive function in the rat model [11]. The medication of CD generally includes AChE inhibitors, donepezil, galantamine, N-methyl-d-aspartate receptor antagonist, and tacrine, all of which are only capable of delaying the decline of cognitive function to some extent, rather than fundamentally change the progression of dementia.

Traditional Chinese medicines (TCMs) have a long history of research, continuously inherited and developed in the period of innovation. TCMs are a combination of several different active substances, which have the advantage of low toxicity and few side effects, leading to the wide application in the study of brain disease [12, 13]. Jiao-tai-wan (JTW), a well-known prescription, was first mentioned in Han's Book on Medicine compiled by MaoHan. The main medical effect of JTW is restoration of normal harmony between heart and kidney, resulting in frequent applications in the treatment of insomnia. JTW contains two types of Chinese traditional medicines: Coptis chinensis (CC) and cinnamon (CIN). The main active ingredients of CC and CIN reduce neurological inflammation and alleviate the symptoms of CD [14–17]. Berberine is a mixture of alkaloids extracted from CC, which has a significant effect on relieving the symptoms of CD [18]. Cinnamic aldehyde, a major active constituent derived from CIN, is distinguished with anti-inflammatory, antioxidative, and neuroprotective effect [19]. In summary, we hypothesized that JTW is effective in neuroprotection.

In the present study, we investigated the effect of JTW and probed its potential mechanisms. Three different doses (2.1g/kg, 4.2g/kg, and 8.4g/kg) of JTW were used to treat the scopolamine- (SCOP-) induced cognitive dysfunction in Kunming mice. Our study found that JTW could significantly alleviate cognitive impairment by improving central cholinergic neurotransmission and prevent SCOP-induced neurodegeneration of the cortex and hippocampus.

## 2. Materials and Methods

### 2.1. Animal and Treatment

Male Kunming mice weighed 21-25g were purchased from Guangzhou University of Traditional Chinese Medicine Experimental Animal Center (Guangzhou, China). The mice were kept in the cage for one week to adapt to the environment and were fed with free food and water. The animals were maintained at 22 ± 2°C temperature, 12-hour light/12-hour dark cycle, and 60% relative humidity throughout the study. The animals were kept in accordance with Guiding Principles for the Care and Use of Laboratory Animals, which was adopted and promulgated by the United States National Institutes of Health. Mice were randomly assigned to six treatment groups (n = 10 per group): control group was given 0.9% saline (CON, n = 10), scopolamine model group (SCOP, n = 10), donepezil group was supplied SCOP 3 mg / kg + ARI 3 mg / kg (DON, n = 10), the low dose has SCOP 3 mg / kg + JTW 2.1 g / kg (JTWL, n = 10), the middle dose contains SCOP 3 mg / kg Kg + JTW 4.2 g / kg (JTWM, n = 10), and the high dose was provided SCOP 3 mg / kg + JTW8.4 g / kg (JTWH, n = 10). Mice were given saline, JTW, donepezil, respectively, for treatment once a day for two weeks by gavage. Donepezil and JTW were given 30 minutes before scopolamine injection. All behavioral tests were performed 30 minutes after the injection of scopolamine. Donepezil and JTW were administered once per day for two weeks throughout the experiment, and scopolamine was given once per day until the end of experiment. All of the animal experiments were approved by the Animal Ethics Committee of Guangzhou University of Chinese Medicine, in accordance with the guide for the animal experiments, clinical studies, and biodiversity rights.

### 2.2. Materials

Scopolamine hydrobromide was dissolved in sterile saline (0.9% NaCl) at a concentration of 0.8 mg/ml. Donepezil was dissolved in distilled water at a concentration of 1 mg/ml. Donepezil was purchased from Shandong Jinan Dexinjia Bio-technology Limited Company (Shandong, China). Scopolamine hydrobromide injection (Guangzhou Baiyun Mountain Mingxing Pharmaceutical Co., Ltd., Guangzhou, China) was purchased from Guangzhou Pharmaceuticals Corporation (Guangzhou, China). Kits used for detection of choline acetyltransferase (ChAT), acetylcholine (Ach), acetylcholinesterase (AChE), reactive oxygen species (ROS), malondialdehyde (MDA), superoxide dismutase (SOD), and catalase (CAT) were purchased from the Nanjing Jiancheng Bioengineering Institute (Nanjing, China). Anti-*β*-actin was purchased from Sigma-Aldrich. Secondary antibodies (horseradish peroxidase conjugated anti-rabbit IgG and anti-mouse IgG) were purchased from Cell Signaling Technology, Inc. Primary antibodies including anti-synaptophysin (SYN), anti-postsynaptic density 95 (PSD95), and anti-brain-derived neurotrophic factor (BDNF) were purchased from Abcam, Inc.

### 2.3. Preparation of JTW

JTW, composed of CC and CIN in proportion to 10: 1, were purchased from the First Affiliated Hospital Pharmacy Room of Guangzhou University of Traditional Chinese Medicine (Guangzhou, China), followed by extraction process carried out at the Laboratory of Pharmacology, Guangzhou University of Traditional Chinese Medicine. The JTW extraction steps were as follows: (a) CIN was immersed for two hours with eight volumes of water, boiled with the extraction of volatile oil equipment for six hours, so that the benzine could be mostly extracted. (b) The dregs and CC were soaked with six volumes of water for two hours, then boiled for five hours. (c) The extraction of liquid was given low temperature heating to get the concentrated liquid. (d) Finally, the volatile oil was mixed into the concentrated liquid.

### 2.4. Morris Water Maze Test

Using Morris water maze test to evaluate the spatial learning and memory ability was mentioned in previous studies [20]. The Morris Water Maze Animal Behavioral Analysis System consists of a black circulation pool (Diameter: 120 cm; Height: 40 cm), a black platform (Diameter: 10 cm), and a recording system. The pool was divided into four quadrants (NE, SE, SW, NW), and different shapes of paper were pasted at the midpoint of the quadrant of the pool wall, which served as the marker for mice to find the platform. The escape platform was placed in the target quadrant center 2 cm below the water. Morris water maze behavior tests to detect the learning and memory abilities of mice were conducted in darkness; therefore curtains should be blocked around the pool. Mice were given a place navigation test for four consecutive days. For each daily trial, there were four sequential training trials, beginning with placing the animal in the water facing the wall of the pool with drop location changing for each trial randomly, followed by the record system starting to record the time. The escape latency was recorded at the end. The mouse would be guided to the platform by the trainer and remained there for 10s if it failed to find the platform within 60 s, whose escape latency would be consequently recorded as 60 s. On the sixth day, the platform was removed and mice were allowed to swim freely in the pool for 60 s. The time of crossing through the original platform position, the time spent in the target quadrant, and the swimming speed were surveyed, which indicated the degree of memory amalgamation.

### 2.5. Y-Maze

The spontaneous alternation of the Y-maze has been reported as an indicator of immediate working memory performance [21]. Y- maze as the horizontal labyrinth is composed of three arms (40×4.5×12 cm, 120°). The maze of floors and walls are made of opaque polyethylene plastic in a black environment. The mice were initially placed in an arm and then placed in the sequence (e.g., 123213, etc.), and the times of each mouse entering the other arms within 5 minutes were manually recorded. Successful alternation was defined as consecutive entries into a new arm before returning to the two previously visited arms. It is necessary to clean up the labyrinth thoroughly and eliminate odor and stains before each test. The maze task was carried out after treatment with scopolamine for 30 minutes. The alternation percentage was defined according to the formula: [(number of alternations)/(total number of arm entries − 2)] × 100 [22].

### 2.6. Novel Object Discrimination (NOD)

The novel object discrimination (NOD) test is a method for learning and memory test, based on the principle that animals have instinct to explore new objects [23]. Experimental device was composed of rectangular black box (60 × 25 × 25cm) and three objects named A, B, C, of which A was identical to B, while the C object was completely different (shape, color) from the A and B. NOD test was divided into two stages: familiarity and testing. In the stage of familiarity, the mouse as well as A and B objects were placed in the box together, adapting for five minutes. After 24 hours for the second phase, object B was replaced with C. The long-term memory recognition (LTMR) was evaluated by the different objects at the same area. The time which the mouse spent distinguishing novel and similar objects could be calculated using the identify index (TNI) = (TN-TF) / (TN + TF) [24, 25]. After each test, the instruments and objects were washed with 70% ethanol to reduce the olfactory hints.

### 2.7. The Fear Conditioning Test (FCT)

The parameters and processes of fear conditioning were conducted by referring to previous studies [26]. The equipment of fear conditioning test includes experimental chamber transparent acrylic room (30*∗*30*∗*30cm), the bottom of the grille floor associated with the shock generator (0.1 ~ 1.0mA shock), the sound generator (broadband or low frequency) connected with a computer. The floor should be cleaned with 70% ethanol before each test and the interior space was white. Additionally, white noise and electrical stimulation were needed during the test. The mouse were placed into the chamber and allowed to explore for 60s, while a 2 Hz pulsation was set (80 dB, 3600 Hz) for 60 seconds and a slight foot shock (0.8 mA, 0.5 s) was given immediately for three times. Consolidations of contextual and auditory fear memory were elucidated, including the number of mice harboring “freezing behavior” in the absence of electrical stimulation of the same environment after 24 hours, the times of occurrences of the behavior to assess the learning and memory. Freezing was defined as completely immobile posture except for those related to respiration.

### 2.8. Measurement of Ach Level and ChAT, AChE Activity

All mice were immediately decapitated under anesthesia at the end of the behavioral tests. Hippocampus and cortex were carefully examined from the brain. All the processes were carried out in ice. Tissue were stored at -80°C quickly. The hippocampus and cortical tissue were homogenized with cold saline. The homogenate was centrifuged at 12,000 x g for 10 minutes at 4°C. Supernatant was collected and total protein concentration was determined using a bicinchoninic acid (BCA) protein assay kit (Nianjing Jiancheng Bioengineering Institute, Nanjing, China) for the assay of the AChE and ChAT activities as well as measuring the level of Ach. Universal Microplate Spectrophotometer (Bio-Rad, Hercules, CA, USA) was used to detect the Ach concentration and the activities of ChAT and AChE according to the manufacturer's instructions.

### 2.9. ROS Production

The hippocampus and cortical tissue were homogenized with cold saline. The homogenate was centrifuged at 12,000 x g for 10 minutes at 4°C. Supernatant was collected to detect the levels of ROS, which was measured by DCFH-DA as a redox sensitive fluorescent dye. DCFH was oxidized to strong green fluorescent substance DCF in the presence of ROS, which has a maximum peak at excitation wavelength of 502nm and emission wave length of 530 and intensity is proportional to intracellular reactive oxygen species.

### 2.10. CAT, MDA, and SOD Assays

The hippocampus and cortical tissue were homogenized with cold saline. The homogenate was centrifuged at 12,000 x g for 10 minutes at 4°C. Supernatant was collected to detect the levels of MDA and the activity of SOD and CAT using Universal Microplate Spectrophotometer (Bio-Rad, Hercules, CA, USA) according to the manufacturer's instructions.

### 2.11. Western Blot Analysis

Brain tissues of hippocampus and cortex were homogenized and lysed in sample buffer (0.5 M Tris/HCl pH 6.8, 50% glycerol, 10% sodium dodecyl sulphate (SDS), 1: 100 inhibitor proteases, and phosphatases cocktail). The lysate was centrifuged at 12,000 ×g for 10min at 4°C, mixed with 1: 4 loading buffer and denatured by boiling at 100°C. The same amount of protein (30 *μ*g) was subjected to SDS-polyacrylamide gel electrophoresis (PAGE) analysis and transferred to PVDF membranes. Thereafter, the membrane was blocked in 5% bovine serum albumin (BSA) that dissolved in Tris-buffered saline-Tween-20 (TBST) for 1 h at room temperature. The membranes containing the protein were incubated with anti-PSD95, anti-SYN, anti-BDNF, and anti-*β*-actin overnight at 4°C. Then the membrane was incubated with horseradish peroxidase conjugated anti-rabbit or anti-mouse for 1 h at room temperature. Finally, the bands on the membrane were visualized using the superenhanced chemiluminescence reagent (ECL; Applygen Technologies Inc., Beijing, China).

### 2.12. Nissl's Staining

Brain paraffin sections were washed in xylene and rehydrated by a graded series of ethanol and double-distilled water. Then these parts were immersed in a Nissl's stain (Nanjing Institute of Bioengineering, Nanjing, China) at room temperature for 5-10 minutes. The slides were rinsed in double-distilled water and dehydrated through 70%, 95%, and 100% gradient alcohol, and removed in xylene. Images were analyzed using optical microscopy and LEICA QWin Plus (Leica Microsystems, Wetzlar, Germany).

### 2.13. Statistical Analysis

Experimental values were given as means ± SEM. SPSS 19.0 statistical software (IBM, Endicott, NY) was evaluated to performed all statistical analysis. Two-way analysis of variance (ANOVA) was applied among the different groups to analyze differences in data for the biochemical parameters, followed by Dunnett's significant post hoc test for pairwise multiple comparisons. Differences were considered as statistically significant at* p*< 0.05.

## 3. Results

### 3.1. HPLC Analysis of JTW

**Figure 1:**
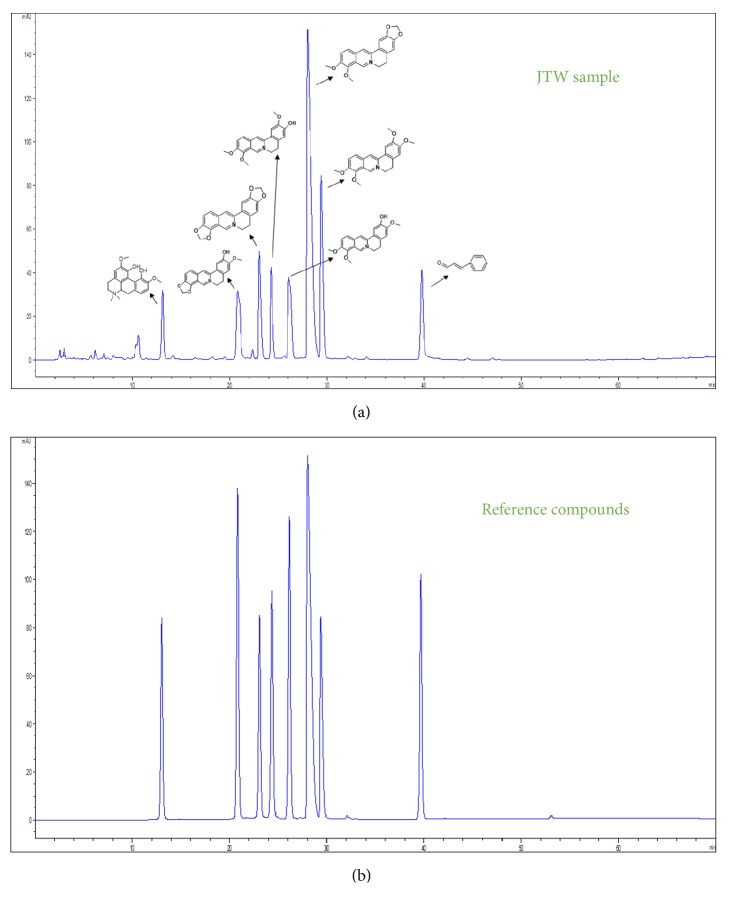
**HPLC of JTW**. HPLC profiles were applied to analyzed chemical standardization of JTW and reference compounds. (**a**) JTW samples; (**b**) reference compounds.

We took advantage of methanol as extract condition to ensure the effective components of JTW. Phenomenex Luna-C18 was indicated as chromatographic condition (250mm*∗*4.6mm, 5*μ*m), detection wavelength, 300nm; flow rate, 1 mL/min. As shown in Figure 1(a), the peak area of JTW in HPLC maps has eight compounds which can be identified as magnoflorine (Peak 1), groenlandicine (Peak 2), coptisine (Peak 3), jatrorrhizine (Peak 4), columbamine (Peak 5), berberine (Peak 6), palmatine (Peak 7), and cinnamaldehyde (Peak 8), by which the berberine was quantified as contents of main composition. The compounds above are compared with reference compounds (in Figure 1(b)).

### 3.2. JTW Improves Learning and Memory of SCOP-Treated Mice

**Figure 2:**
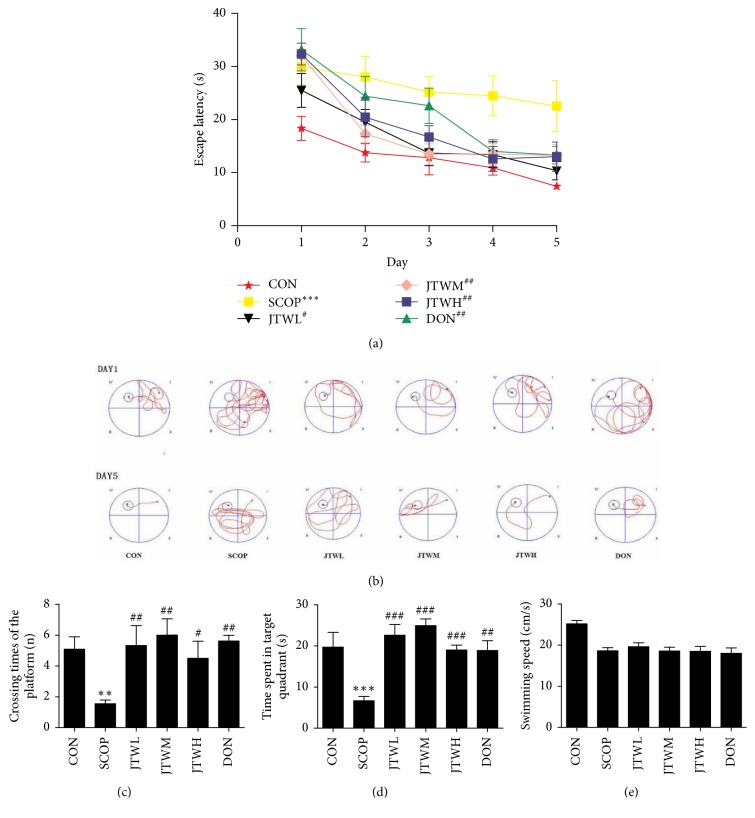
**Effects of JTW prevents SCOP-treated learning and memory impairments by Morris water maze test**. (**a**) Escape latency of five consecutive days test. (**b**) The swimming paths of respective groups on first and fifth day. (**c**) Crossing times of the target platform in the probe trial. (**d**) Time spent in the target quadrant in the probe trial. (**e**) The swimming speed in the probe trial. JTWL: Jiao-tai-wan (2.1g/kg/d); JTWM: Jiao-tai-wan (4.2g/kg/d); JTWH: Jiao-tai-wan (8.4 g/kg/d); DON: donepezil. Experimental values were expressed as mean ± SEM (*n* = 16 per group). ^*∗*^*P* < 0.05, ^*∗∗*^*P* < 0.01, ^*∗∗∗*^*P* < 0.001 versus CON group; ^#^*P* < 0.05, ^##^*P* < 0.01, ^###^*P* < 0.001 versus SCOP group.

In order to investigate whether JTW could alleviate the SCOP-treated spatial-working, learning and memory impairment, four types of behavioral tests were conducted: Morris water maze test, Y-maze, novel object discrimination, and fear conditioning test. As shown in Figures 2(a) and 2(b), within five training days, the mice progressively reduced the time to find hidden platforms. Compared with the control group, the SCOP intraperitoneal injection group had an extremely long time to find a hidden platform. However, the preprotective effect of Jiao-tai-wan and donepezil led to significantly shortened escape latency in JTW low-dose, middle-dose, and high-dose group as well as donepezil group when compared to the SCOP group. On the sixth day, the experiment of estimating their spatial-working memory was conducted, the platform was removed and the mice were allowed to swim freely. A longer latency was observed in the SCOP group, with relatively less time stayed at the target quadrant and fewer times crossing the position of the removed platform (*p *<0.001, Figures 2(c), 2(d), and 2(e)). In the JTW and the DON groups, the time for the mice to stay in the target quadrant and the number of crossing platforms were prominently increased. The swimming speed decreased remarkably in the SCOP group, but there was no significant difference among the control, JTW, and DON groups.

**Figure 3:**
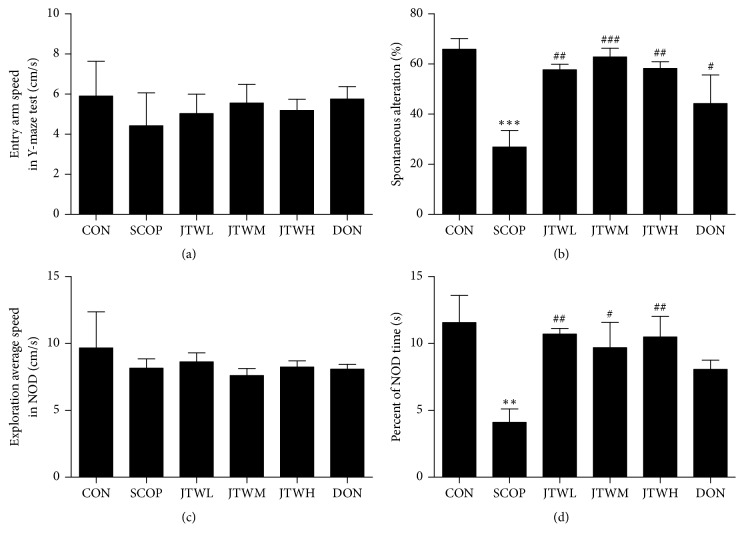
**Effects of JTW prevents SCOP-treated learning and memory impairments by Y-maze and novel object discrimination**. (**a**) Entry arm speed in Y-maze test. (**b**) Changes in percentage of spontaneous alternation in different groups. (**c**) Exploration average speed in NOD. (**d**) Novel object preference index. JTWL: Jiao-tai-wan (2.1g/kg/d); JTWM: Jiao-tai-wan (4.2g/kg/d); JTWH: Jiao-tai-wan (8.4 g/kg/d); DON: donepezil. Experimental values were expressed as mean ± SEM (*n *= 16 per group). ^*∗*^*P* < 0.05, ^*∗∗*^*P* < 0.01, ^*∗∗∗*^*P* < 0.001 versus CON group; ^#^*P* < 0.05, ^##^*P* < 0.01, ^###^*P* < 0.001 versus SCOP group.

Then, the effect of JTW on the SCOP-induced deficit was assessed using the Y-maze test, a well-known method for working memory (Figures 3(a) and 3(b)). Entry arm speed in Y-maze test was shown in Figure 3(a); the entry speed in JTW-treated group was decreased than control group. A remarkable increase of spontaneous alternation index was observed in the vehicle control group, in contrast to the SCOP group in Figure 3(b) (*p*<0.01,* p*<0.001). The spontaneous alternation index of the JTW (low-dose, middle-dose, high-dose) and DON groups have significant improve compared to the SCOP group.

In order to evaluate the impact and improvement of JTW on behavioral disorders, the NOD was applied to determine the ability of mice to distinguish between complex setting and different novel objects. The exploration average speed in JTW (low-dose, middle-dose, high-dose) groups was decreased than control group in NOD. However, the percent of NOD time of mice to explore novel objects was significantly increased in JTW (low-dose, middle-dose, high-dose) groups (Figures 3(c) and 3(d)).

**Figure 4:**
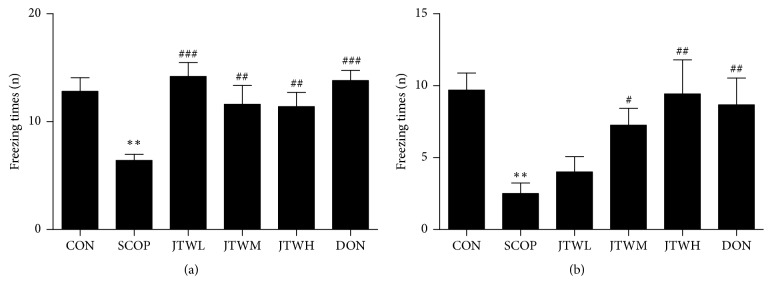
**Effects of JTW prevents SCOP- treated learning and memory impairments by fearing condition test**. (**a**) 24 hours after the first FCT is completed, the freezing times of the mouse displayed under the same environment and light stimulation but no electrical stimulation. (**b**) The freezing times of the mouse displayed at the first FCT under light and electrical stimulation. JTWL: Jiao-tai-wan (2.1g/kg/d); JTWM: Jiao-tai-wan (4.2g/kg/d); JTWH: Jiao-tai-wan (8.4 g/kg/d); DON: donepezil. Experimental values were expressed as mean ± SEM (*n *= 16 per group). ^*∗*^*P* < 0.05, ^*∗∗*^*P* < 0.01, ^*∗∗∗*^*P* < 0.001 versus CON group; ^#^*P* < 0.05, ^##^*P* < 0.01, ^###^*P* < 0.001 versus SCOP group.

The fear conditioning test (FCT) was applied to evaluates ability to remember fear based on the freezing behavior number of times in mice, differences were observed between the control and the SCOP group in both the contextual and the cued recall paradigms, with less freezing times presented in the SCOP group (*p* < 0.001,). In contrast, the JTW and DON groups have more freezing times (Figures 4(a) and 4(b)).

### 3.3. JTW Improves the Cholinergic Nerve System in SCOP-Treated Mice

**Figure 5:**
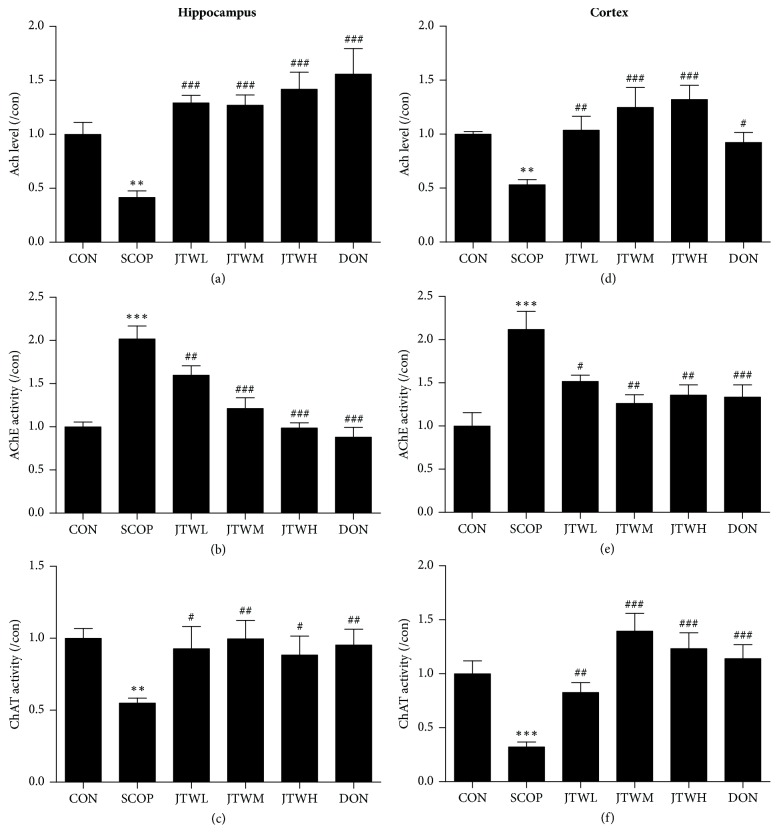
**Effects of JTW improves the cholinergic nerve system in SCOP-treated Mice**. The supernatant of hippocampus and cortex homogenate was used for the assay. The level of Ach and the activities of AChE and ChAT in hippocampus (**a**,** b** and** c**). The level of Ach and the activities of AChE and ChAT in cortex (**d**,** e** and** f**). JTWL: Jiao-tai-wan (2.1g/kg/d); JTWM: Jiao-tai-wan (4.2g/kg/d); JTWH: Jiao-tai-wan (8.4 g/kg/d); DON: donepezil. Experimental values were expressed as mean ± SEM (*n *= 16 per group). ^*∗*^*P* < 0.05, ^*∗∗*^*P* < 0.01, ^*∗∗∗*^*P* < 0.001 versus CON group; ^#^*P* < 0.05, ^##^*P* < 0.01, ^###^*P* < 0.001 versus SCOP group.

In order to illuminate the potential mechanism of JTW in ameliorating cognition deficiency in SCOP-mice, the activities of cholinergic marker enzymes were detected. In the group of JTW and DON, the level of acetylcholine (Ach) was significantly improved in both hippocampus and cortex. However, Ach level of SCOP group was evidently declined (Figures 5(a) and 5(d)). The acetylcholinesterase (AChE) activity was increased by scopolamine in the hippocampus and cortex (*p* < 0.001,* p* < 0.01, shown in Figures 5(b) and 5(e)), while it was significantly decreased by the treatment with JTW and DON groups. As shown in Figures 5(c) and 5(f), the activity of choline acetyltransferase (ChAT) were significantly increased with the application of JTW and DON, whereas they were sharply decreased in the SCOP-treated group in both hippocampus and cortex. In summary, JTW can protect cognitive deficits by affecting cholinergic nervous system.

### 3.4. JTW Improves the Oxidative Stress Status in SCOP-Treated Mice

**Figure 6:**
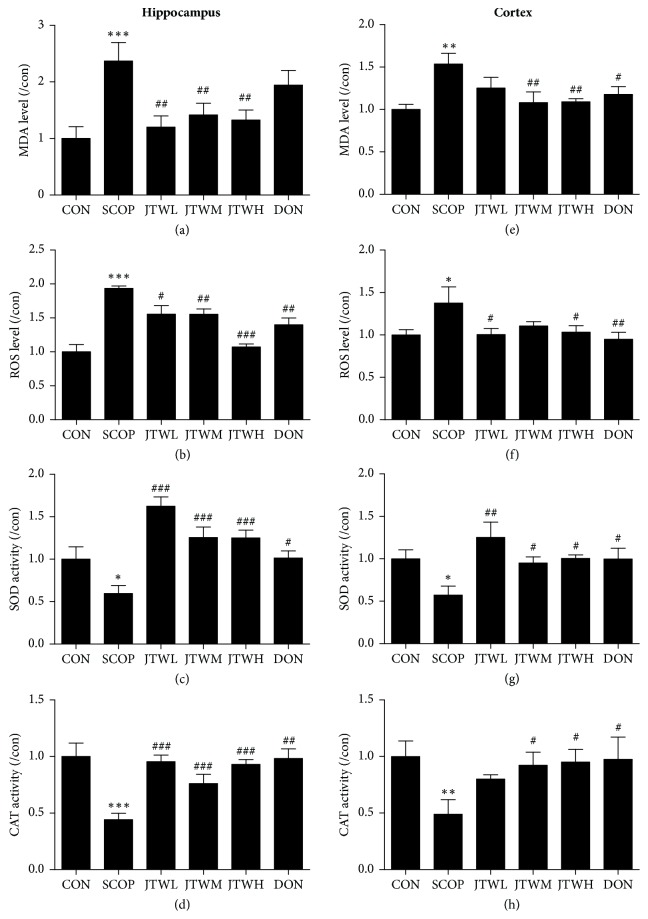
**Effects of JTW improves the Oxidative Stress Status in SCOP-treated Mice**. The supernatant of hippocampus and cortex homogenate was used for the assay. The level of MDA, ROS and the activities of SOD and CAT in hippocampus (**a, b, c, and d**). The level of MDA, ROS and the activities of SOD and CAT in cortex (**e, f, g, and h**). JTWL: Jiao-tai-wan (2.1g/kg/d); JTWM: Jiao-tai-wan (4.2g/kg/d); JTWH: Jiao-tai-wan (8.4 g/kg/d); DON: donepezil. Experimental values were expressed as mean ± SEM (*n *= 16 per group). ^*∗*^*P* < 0.05, ^*∗∗*^*P* < 0.01, ^*∗∗∗*^*P* < 0.001 versus CON group; ^#^*P* < 0.05, ^##^*P* < 0.01, ^###^*P* < 0.001 versus SCOP group.

Oxidative stress state can be used to determine the state of hippocampus and cortex of SCOP-treated mice. Scopolamine had a robust effect on in the hippocampus and cortex. Consequently, increased levels of MDA and ROS (Figures 6(a), 6(b), 6(e), and 6(f)), and suppressed activities of SOD and CAT (Figures 6(c), 6(d), 6(g), and 6(h)) were observed in the SCOP group, JTW and DON groups respectively. Obviously, the MDA and ROS levels were ameliorated by JTW and DON, which were able to increase the activity of SOD and CAT at the same time. In contrast, the SCOP group did not improve activities of SOD and CAT and levels of MDA and ROS.

### 3.5. JTW Improves the Neurodegeneration in SCOP-Treated Mice

**Figure 7:**
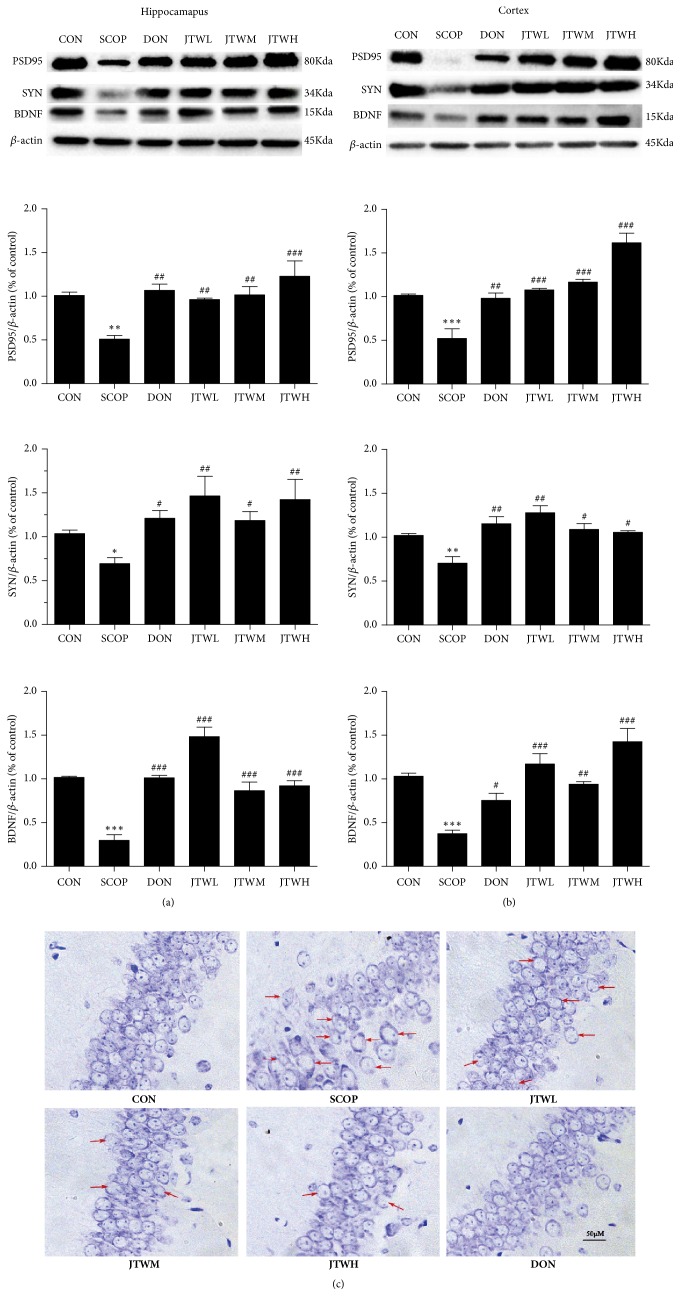
**JTW improves the neurodegeneration in SCOP-treated mice**. PSD95, SYN, BDNF protein levels are detected by Western Blotting in hippocampus (**a**) and cortex (**b**). (**c**) Nissl's staining in parietal hippocampus. Scale bar: 50 *μ*m. JTWL: Jiao-tai-wan (2.1g/kg/d); JTWM: Jiao-tai-wan (4.2g/kg/d); JTWH: Jiao-tai-wan (8.4 g/kg/d); DON: donepezil. Experimental values were expressed as mean ± SEM (*n *= 16 per group). ^*∗∗∗*^*P* < 0.001 versus CON group; ^#^*P* < 0.05, ^##^*P* < 0.01, ^###^*P* < 0.001 versus SCOP group.

As shown in Figures 7(a) and 7(b), the expression of brain-derived neurotrophic factor (BDNF), postsynaptic density 95 (PSD95) and synaptophysin (SYN) as neurotrophic factors were sharply decreased in the SCOP-treated mice. Due to the treatment of JTW and DON, the protein returned to normal levels and were fully expressed. Nissl's staining was applied to hippocampus as a further nerve cell test method (Figure 7(c)). In the hippocampal subfield of SCOP-treated mice, the neurons were significantly shrunken, irregularly arranged, and weakly stained, which indicated that neurons were diffusely deteriorated or dead and great quantity Nissl bodies were lost in these neurons. Conversely, the neurons exhibited regularly arrange, normal morphology and were deeply stained in the JTW and DON groups. To sum up, JTW could ameliorate neurodegeneration in SCOP- treated mice.

## 4. Discussion

In this study, JTW was used to evaluate the mechanism of cognitive dysfunction using Scopolamine-treated models. We found that JTW dose (2.1 g / kg, 4.2 g / kg, 8.4 g / kg) slightly affect the ability of behavior, but could relieve SCOP- treated impairment of learning and memory in Kunming mice. Meanwhile, JTW could significantly ameliorate the central cholinergic nerve conduction and the injure caused by oxidative stress. In addition, JTW could alleviate neurodegeneration in the hippocampus and cortex.

The damage of cholinergic system in brain is one of the classic hypotheses of the development and progression of AD [27]. Scopolamine is an antagonist of M cholinergic receptor. By blocking the binding of acetylcholine (ACh) with M receptor, it inhibits the transmission of brain information and interferes with the formation of short-term memory, thus simulating the damaged [28] in the AD. Cholinergic system and interfering with the coding and extraction process of spatial-working memory and reference memory respectively [29–31]. Donepezil has potent acetylcholinesterase inhibition, strong selectivity and long acting time [32]. Oral administration of donepezil can inhibit the hydrolysis of acetylcholine by cholinesterase in a dose-dependent manner. Due to the high selectivity of donepezil to the acetylcholinesterase in the central nervous system, the inhibition of enzyme activity for a long time and no peripheral action, it can improve the concentration of acetylcholine in the central nervous system, especially in the cerebral cortex and basal ganglia synapses, thus improving the cognitive function. Donepezil can prevent and control primary neuronal damage by adjusting metabolism and restoring function [33–35].

Berberine is an acetylcholinesterase inhibitor [36], whose major active constituents were extracted from traditional Chinese medicine Coptis chinensis and other plants [37], which has been used in the field of Chinese medicine and has a long-term clinical efficacy [38]. In the central nervous system diseases, Berberine has a neuroprotective function through its anti-inflammatory, anti-oxidative and anti-neuronal apoptosis pharmacological properties [39–41]. After easy penetration through the blood-brain barrier, berberine is transported into neurons with a slow rate of elimination, indicating that it has a direct effect on neurons and is accumulated in the hippocampus [42]. Moreover, recent researches have indicated that berberine exerts neuroprotection in cerebral ischemia and Alzheimer's disease [43, 44]. Based on previous studies, the NF-kB signaling pathway was found as the pathogenesis of AD [45, 46]. Wenbo He [47] through the application of berberine to APP/PS1 transgenic mice, found that berberine can inhibit the activation of NF-kB signaling pathway to delay the effect of oxidative stress and neurological inflammation, thereby effectively attenuated cognitive dysfunction. In addition, berberine can stimulate GSH synthesis and promote the activity of several anti-oxidative enzymes to protect cells from oxidative damage [48, 49]. Shaktipal Patil [50] used berberine to treat ethanol-induced memory impairment in rats. The model was triggered by oxidative stress and cholinergic dysfunction. The result found that berberine can improve oxidative stress and cholinesterase activity, berberine has improved memory dysfunction caused by abnormal oxidative stress and cholinergic function. Cinnamaldehyde, a main component of traditional Chinese medicine cinnamon, is biologically active as anti-inflammatory and anti-oxidative agent [51, 52], evidenced by inhibited the activity of inflammatory factors NF-kb, Nosynthase, COX-2 [53] and suppressed release of noradernaline in guinea-pig ileum myenteric nerve terminals [54, 55]. In the experiment, Yang D [19] found cinnamaldehyde have anti-oxidation and anti-inflammatory effects, and it had repair and protection effects on the injured neurons of rat dorsal ganglion. To sum up, the main components of JTW can improve oxidative stress, neuroinflammation, cholinergic abnormalities and other factors related to cognitive function.

Cognitive dysfunction as a moderate state between normal cognition and dementia, which has become a major public health issue due to its high socioeconomic status [56] which is characterized as the gradual loss of learning ability and cognitive function, as well as memory impairment, which are considered as typical symptoms of AD [57, 58]. A vast number of experimental studies have suggested that cholinergic neurotransmission dysfunction in the cerebral hippocampus and cortex plays an important role in cognitive impairment [4]. The central nervous system of AD patients shows abnormalities in cholinergic function are closely related to cognitive dysfunction [59]. Some studies [7, 60] have found that when treating cognitive dysfunction, the use of drugs that activate the cholinergic system can promote learning and memory functions in animals. Scopolamine (SCOP) as inducing amnesia in mammals, which is a non-selective anti-muscarinic that penetrates the blood-brain barrier (BBB) and produces a model similar to early AD symptoms [61, 62]. SCOP inhibits central cholinergic neurons by blocking two major systems of the forebrain cholinergic activity (nuclei basalis-cerebral cortex and septohippocampal pathways), resulting in accurate, repeatable and transient memory damage to normal humans and healthy animals [63, 64]. We introduced the following behavior tests to observe the SCOP-induced cognitive dysfunction model: Morris water maze test, Y-maze, NOD and FCT to measure the memory deficits of the SCOP-treated mice. Results show that the SCOP group which accepted JTW treatment have obvious ameliorated effect on learning ability and cognitive function, as well as memory impairment. The SCOP group have a longer latency, a few of crossing times of the platform and time spent in target quadrant, cognitive function have not been improved.

Some studies have also reported that the treatment of cholinergic neurotransmitters is the most promising treatment for cognitive dysfunction caused by SCOP [65]. Acetylcholine (ACh) [66] is considered to be the most important neurotransmitter involved in the regulation of cognitive functions. After releasing from the presynaptic neuron, Ach, the neurotransmitter, was gathered into the synaptic cleft, followed by binding to the Ach receptors on the postsynaptic membrane, and the signal from the nerve was relayed during the process [67]. The termination of the signal transmission was conducted by hydrolyzation of ACh located on the postsynaptic membrane [68]. AChE, one of the most important enzyme in the family serine hydrolase involved in the hydrolytic cleavage of Ach, depletes the levels of ACh involved in memory and learning [69]. Recent studies have found that AChE increased its activity is a major cause of neurodegenerative diseases. Through electron emission tomography, AChE was severely increased in the forebrain of AD patients [70, 71]. ChAT is a key part of the synthesis of cholinergic neurons, which participates in Ach synthesis. The protein express and activity of ChAT decreased at early stages of AD and cognitive impairment [72, 73]. The decline in the activity of choline acetyltransferase (ChAT) and acetylcholine esterase (AChE), as well as the release amount of acetylcholine (ACh) often appear in the SCOP-treated mice [74]. After inspection of the cholinergic system in SCOP-treated mice in the study, we concluded that the level of ACh and the activity of ChAT were increased while the activity of AChE was decreased in the JTW groups compared with the SCOP group.

Brain tissue is most vulnerable to the harmful effects of reactive oxygen species. Reactive oxygen species (ROS) may lead to the process of lipid peroxidation in neuron degeneration, which is most prominent in the central cholinergic pathway. Because the ROS has a high oxygen consumption rate and can reduce the capacity of the antioxidant defense system [75, 76]. Some research proposed that ROS generated by oxidative stress is one of the pathogeneses of dementia, including AD and CD. Yuan Hu [77] have found that when the supply of oxygen to the brain was low, ROS production, which promote membrane lipid oxidation were extremely increased, DNA, and essential proteins, leading to neuronal apoptosis and cognitive dysfunction. Malondialdehyde (MDA) was used as an end product of lipid peroxidation and commonly be used to demonstrate the occurrence of increased peroxidative stress [78], which was mainly distributed in various parts of the brain, such as the cortex, the hippocampus, the striatum, and the whole brain [79]. At the same time, the degree of oxidative stress determines the severity of this cognitive impairment. Superoxide dismutase (SOD) is an endogenous antioxidant enzyme, when the superoxide-oxidase level was exhausted, the resulting superoxide can directly cause cell damage [80]. Catalase as a parameter of oxidative stress in brain tissue and was used to study homogenates of cognitive dysfunction in animals [81]. Studies reported that when different parts of the brain were exposed to various chemicals, it causes a significant drop in CAT and causing cognitive impairment [80]. The activities of the antioxidant enzymes CAT and SOD were significantly diminished due to SCOP- treated cognitive impairment [82]. In the study, robustly increased ROS and MDA levels and stifled activities of SOD and CAT were detected in the SCOP- treated group. Furthermore, the ROS and MDA levels were inhibited while the SOD and CAT activities were increased in both hippocampus and cortex in the JTW-treated groups.

Synapses, phosphoproteins, was associated with synaptic vesicles participated in neuronal development and neurotransmitter release [83, 84]. Many research have reported that impaired cognitive ability, learning ability and memory are alternately associated with synapses [85]. Postsynaptic density protein 95 (PSD95), mainly in the hippocampus and prefrontal cortex, are closely related to cognitive disorders [86]. Synaptophysin (SYN) is a protein that has an important influence on cognitive function and is located in the presynaptic vesicle [86]. Brain-derived neurotrophic factor (BDNF) is one of the most important brain - derived neurotrophic factors in the brain. It is mainly involved in the response to learning, exercise, memory, and external stress [87]. BDNF is produced during the process of synaptic pruning and apoptosis, and regulates neurotransmission through the involvement of synapses, depending on the presence of synapses [88–90]. Increased expression of BDNF can reduce neuronal synaptic damage in nervous system diseases [91]. In this study, there were certain neuronal damages. In the JTW and DON groups, the low expressions of PSD95, SYN and BDNF were reinstated. In addition, Nissl's staining was applied to prove the neuroprotective effect of JTW. The above results demonstrated that the protective effect of JTW was able to inhibit SCOP- treated neurodegeneration in the hippocampus and cortex.

## 5. Conclusion

We validated that the treatment of JTW can effectively promote the ability of spatial recognition, learning and memory, and the memory ability of fresh things of SCOP-treated mice. Then, the activity of acetylcholinesterase (AChE) was effectively decreased; the activity of choline acetyltransferase (ChAT) and concentration of acetylcholine (Ach) were improved after JTW treatment in SCOP-treated mice. In addition, JTW effectively ameliorated oxidative stress because of decreased the levels of malondialdehyde (MDA) and reactive oxygen species (ROS) and increased the activities of superoxide dismutase (SOD) and catalase (CAT) in hippocampus and cortex. At last, JTW promote the expressions of neurotrophic factors including postsynaptic density protein 95 (PSD95) and synaptophysin (SYN) and brain-derived neurotrophic factor (BDNF). As part of multi-target strategies, JTW might be a potential anti-AD drug. However, further mechanisms and clinical trials are warranted for a better understanding and clinical practice of JTW.

## Data Availability

All original data in the manuscript are available from the author.
